# *Saccharomyces cerevisiae*‐Fermented Salmon Placental Protein Inhibits Muscle Loss in a Mouse Model of Sarcopenia

**DOI:** 10.1002/fsn3.71370

**Published:** 2025-12-29

**Authors:** Jung‐Chang Lee, Tae‐Joong Lim, Hye‐Min Lee, Sung‐Moon Jun, Kyung‐Mi Lee

**Affiliations:** ^1^ Department of Biochemistry and Molecular Biology, College of Medicine Korea University Seoul Republic of Korea; ^2^ Nature Pharm Corp Gimpo‐si Gyeonggi‐do Republic of Korea

**Keywords:** branched‐chain amino acid, C2C12, *Saccharomyces cerevisiae* ‐fermented salmon placental protein (F‐Sal), sarcopenia

## Abstract

Addressing sarcopenia and frailty in aging populations is crucial for improving quality of life and reducing healthcare dependence among these patients. Although no specific drugs have been approved for treating sarcopenia, protein supplements rich in branched‐chain amino acid (BCAA) have been reported to increase muscle mass and function in elderly individuals. Fermentation of salmon placenta protein is an effective approach to improve BCAA content compared to previous sarcopenia‐related protein supplements. Therefore, this study aimed to evaluate BCAA content through salmon placenta protein (Sal) fermented with *Saccharomyces cerevisiae* (F‐Sal), analyze the expression of muscle‐forming genes in C2C12 myoblasts in vitro, measure the weight gain of quadriceps femoris and gastrocnemius muscle, measure the volume and thickness of gastrocnemius muscle, and measure the expression of biomarkers related to sarcopenia in quadriceps femoris and gastrocnemius muscle in a mouse model of sarcopenia. Here, we report for the first time that salmon placental extracts (Sal) fermented with *S. cerevisiae* (F‐Sal) exhibit drastically elevated levels of BCAA, including leucine, isoleucine, and valine, and ameliorated the conditions of sarcopenia in both in vitro and in vivo sarcopenia models. When the addition of Sal or F‐Sal to cultured C2C12 myoblasts with dexamethasone‐induced sarcopenia, the expression level of the muscle‐forming gene MyoD was significantly increased in F‐Sal compared to Sal (*p* < 0.01), and the expression level of the muscle‐degrading gene MuRF1 was significantly decreased in F‐Sal compared to Sal (*p* < 0.01). In a mouse model of sarcopenia, oral administration of F‐Sal for 14 days following dexamethasone treatment increased muscle weight and the expression of muscle‐forming genes while mitigating muscle loss and reducing the expression of muscle‐degrading genes in both the quadriceps femoris and gastrocnemius muscles. These findings provide a therapeutic basis for treating muscle weakness in patients with end‐stage disease as well as sarcopenia‐related muscle conditions in elderly by improving BCCA content with *S. cerevisiae* ‐fermented salmon placental protein (F‐Sal).

## Graphical abstract

*Saccharomyces cerevisiae* ‐based fermentation significantly increases the branched‐chain amino acid (BCAA) levels in salmon placental protein fourfold. In cell and animal models, F‐Sal improves muscle function and promotes muscle‐forming gene expression. F‐Sal exerts anti‐inflammatory effects and reduces the expression of markers linked to muscle degradation, making it a promising treatment (including the new opportunities in functional food development) for age‐related muscle loss. In vivo efficacy of Sal and F‐Sal in a dexamethasone‐induced mouse model of sarcopenia.

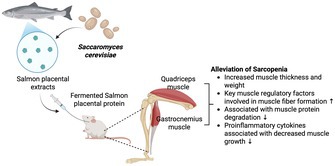

## 1 Introduction

Sarcopenia is an aging‐related disease that causes a gradual reduction in muscle mass, a decrease in muscle strength, and a decrease in muscle function. Recent studies have shown that sarcopenia causes a rapid decrease in the basal metabolic rate of muscle mass, which increases insulin resistance and promotes the oneset of type 2 diabetes, and increases the risk of hypertension and cardiovascular disease (Quan et al. 2023). And in a study of people with sarcopenia, it was reported that the risk of developing Alzheimer's dementia increased by up to 50% (Beeri et al. 2021). Therefore, sarcopenia is closely related to a decline in the quality of life and a shortened healthy lifespan, and is emerging as a very important aspect of the health of the elderly population. Current treatments for sarcopenia mainly include nutritional and exercise interventions (Arnarson et al. 2013; Bauer et al. 2015; Yoshimura et al. 2016; Naseeb and Volpe 2017), but the clinical evidence of their efficacy is limited. Although no specific drugs have been approved for the treatment of sarcopenia, several pharmacological interventions, including anabolic growth hormone, steroids, and their analogs, have been shown to improve this condition in elderly individuals (Kwak and Kwon 2019). However, the effectiveness of pharmacological interventions for treating sarcopenia is limited and is often associated with adverse effects (Morley 2016).

Recent studies have shown that supplementation with branched‐chain amino acid (BCAA), such as leucine, isoleucine, and valine, may help prevention of sarcopenia (Volpi et al. 2003), and low BCAA levels have been associated with loss of muscle mass, decline of muscle function, and muscle weakness in community‐dwelling older adults (ter Borg et al. 2019). Placental extracts, known to be high in BCAA, have been reported to have antioxidant and anti‐inflammatory effects (Banerjee et al. 1992; Togashi et al. 2000; Park et al. 2010; Sur et al. 2013; Philipps et al. 1978). For example, human placental extract (HPE) has been shown to significantly inhibit the production of nitric oxide, tumor necrosis factor‐α, and cyclooxygenase‐2 in lipopolysaccharide‐stimulated RAW264.7 macrophages (Lee et al. 2011). Salmon placental proteins, often collectively called marine placental extract, are derived from salmon roe; this extract is freshly obtained from salmon ovaries and marketed in Japan as “marine placenta” (Kim et al. 2015). Several studies have documented the differing compositions of fresh salmon roe across various species (Moriya et al. 2007; Bekhit et al. 2009; Ahmmed et al. 2021). Placental extracts high in BCAA levels have been shown to stimulate fibroblast proliferation and collagen production, which form the structural framework of animal tissues in vitro (Pan et al. 2017). Notably, fermentation has been shown to increase the nutritional value of salmon roe and yield health‐promoting and functional products (Bekhit et al. 2018). For example, salt‐treated fermentation of Chinook ( *Oncorhynchus tshawytscha* ) salmon eggs improved polyunsaturated fatty acid (PUFA) content and reduced cholesterol content (Bunga et al. 2023). Interestingly, the content of branched‐chain amino acid (BCAA) increased during the fermentation of mandarin fish ( *Siniperca chuatsi* ) with Lactobacillus sake SMF‐L5 (Zhou et al. 2023). *Saccharomyces cerevisiae* (baker's yeast) is a common model organism with fermentation capacity, and the metabolic pathways by which it converts amino acids through various nitrogen compounds are well known (Ljungdahl and Daignan‐Fornier 2012). However, there is no documented evidence regarding the amino acid changes during the *S. cerevisiae* ‐mediated fermentation of salmon placental protein.

Therefore, in this study, to maximize the therapeutic effect of sarcopenia compared to existing BCAA supplements, we fermented salmon placenta protein using four microorganisms: *S. cerevisiae* , *Aspergillus oryzae*, *Bacillus subtilis* , and *Lactobacillus plantarum* , and compared and analyzed the BCAA content. Among the four microorganisms, salmon placental protein fermented with *S. cerevisiae* (F‐Sal) in particular served as a promising method to maximize BCAA content, and thus its therapeutic potential for improving sarcopenia was explored in both in vitro and in vivo models. Using a mouse dexamethasone (DEX)‐induced sarcopenia model, we confirmed that *S. cerevisiae* ‐fermented salmon placental protein ameliorates sarcopenia, offering new insights into muscle aging‐related diseases. Furthermore, considering the nutritional and metabolic significance of F‐Sal, establishing an optimization system for the BCAA content of F‐Sal is essential for exploring new opportunities in functional food development. Therefore, this study aimed to develop a fermentation method for salmon placenta protein using *S. cerevisiae* to maximize BCAA content, restore muscle morphology and expression of muscle‐forming genes within cells, and measure the expression of biomarkers that suppress muscle loss in the quadriceps femoris and gastrocnemius muscles in a sarcopenia mouse model.

## 2 Materials and Methods

### 2.1 Salmon Placenta and Fermented Salmon Placenta

To produce salmon placental protein (Sal), Alaskan salmon roe (Maruha Nichirio, Japan) was first dried at 58°C for 15 h (WFO‐400, Tokyo Rikakikai, Japan) and then pulverized in a mixer (SHMF‐3500TG, Hanil, Korea). Subsequently, 95% ethanol was added to the dried salmon roe powder in a 3:1 ratio by weight. Lipids were then removed via shaking at 160 rpm in a 60°C incubator (BF‐150SIR‐4R; BioFree, Korea) for 15 h. The extract was then vacuum filtered through a 1‐μm filter (No. 53 filter paper; Hyundai Micro, Korea), after which the solid residue that remained on the filter paper was recovered. This residue was then dried at 58°C for 15 h to prepare the raw Sal material.

To prepare fermented salmon placental protein, 200 g of the raw Sal was mixed with distilled water to a total volume of 1 L. The mixture was stirred for 3 min and then allowed to settle. After separation, the upper layer (approximately 150 mL) was collected, and its volume was measured. The collected upper layer was diluted 1:1 with distilled water. This process was repeated three times, after which the final pellet layer, recovered by removing the upper layer, was subjected to fermentation. For the fermentation, *S. cerevisiae SRCM100587* (Microbial Institute for Fermentation Industry, Sunchang, Korea), *A. oryzae SRCM102021* (Microbial Institute for Fermentation Industry, Sunchang, Korea), *B. subtilis* *SRCM100761* (Microbial Institute for Fermentation Industry, Sunchang, Korea), and *L. plantarum* (isolated directly from kimchi) strains were individually prepared in 100 mL volumes in media containing 2% glucose, 1% yeast extract, and 2% peptone (pH 7.0) as inoculum. The media were sterilized at 121°C for 15 min and inoculated with 0.1% inoculum. Precultivation was carried out for 15 h at 30°C and 160 rpm. Prior to the main cultivation, the yeast extract concentration and the peptone concentration in the cultivation medium (salmon meal mixture) were adjusted to 0.1% and 0.2%, respectively, and glucose was added to a concentration of 1%. Sterilization was then performed at 121°C for 30 min. *S. cerevisiae* , *A. oryzae* *SRCM102021*, *B. subtilis* *SRCM100761*, and *L. plantarum* were subsequently inoculated at a ratio of 10% (v/v) in the main fermentation broth, and fermentation was conducted for 2 days at 30°C and 160 rpm. The F‐Sal powder obtained following the *S. cerevisiae* fermentation was sterilized at 100°C for 30 min and then freeze‐dried at −50°C ~ 60°C for 48 h (FDS8508; IlsinBiobase, Korea) to obtain the final product.

### 2.2 Amino Acid Composition and Content Analysis

The freeze‐dried Sal and F‐Sal samples were reconstituted in phosphate‐buffered saline (PBS) at a concentration of 5% (pH 7.4), followed by centrifugation at 4000 rpm for 10 min. The resulting supernatant was filtered through a 0.2‐μm filter, and the contents of free and bound amino acids were assessed. Automated amino acid analyses were conducted with an Agilent 1290 high‐performance liquid chromatography (HPLC) system (Agilent Technologies), which allows the precise quantification and identification of the amino acids present in the samples (Carducci et al. 1996). To complement the amino acid analysis, the proteins within F‐Sal and Sal were identified with liquid chromatography–mass spectrometry (LC–MS).

### 2.3 C2C12 Cultures and Cytotoxicity Measurements for the Raw Materials

C2C12 cells were purchased from the American Type Culture Collection (ATCC; Manassas, VA, USA) for the in vitro cell experiments. The C2C12 cells were cultured in Dulbecco's modified Eagle's medium (DMEM) supplemented with 10% fetal bovine serum (FBS) and 1% penicillin–streptomycin (P/S, 100 U/mL penicillin, 100 μg/mL streptomycin) at 37°C in a humidified atmosphere containing 5% CO_2_ for 5 days (Park et al. 2020). To assess the cytotoxicity of Sal and F‐Sal to the C2C12 cells, the cells were seeded in a 96‐well plate at a density of 5000 cells per well and cultured for 24 h. Then, medium containing Sal or F‐Sal at concentrations ranging from 5, 10, 15, 30, 60, 125, 250, 500 μg/mL was added to the wells, and the cells were cultured for an additional 24 h. Following this incubation period, C2C12 cell proliferation was evaluated by measuring the absorbance at 490 nm via a Cell Counting Kit‐8 (CCK‐8) from Dojindo Laboratories (Kumamoto, Japan).

### 2.4 Myotube Differentiation and the Induction of Sarcopenic Conditions

C2C12 cells were cultured in DMEM supplemented with 10% fetal bovine serum (FBS) and 1% P/S and then induced to differentiate. To induce myotube formation, the cells were cultured in DMEM supplemented with 2% horse serum and 1% P/S at 37°C in a 5% CO_2_ atmosphere (Sandri et al. 2004). To confirm the efficacy of Sal and F‐Sal in preventing sarcopenic conditions in differentiated myotubes, cells were divided into a control group and experimental groups and incubated in DMEM supplemented with 1% P/S, with or without 100 μM dexamethasone (DEX). After treatment, the groups were treated with Sal or F‐Sal at nontoxic concentrations for 24 h. The cells were observed under a Nikon light microscope (Nikon, Tokyo, Japan) for evaluation of morphological changes indicative of sarcopenia.

### 2.5 Animal Experiments

Male C57BL/6 mice aged 6 weeks were obtained from Samtako Bio Korea Co. Ltd. (Gyeonggi‐do, Korea) for in vivo experiments. The experimental animals were acclimated for 1 week under the following environmental conditions: temperature, 24°C ± 2°C; relative humidity, 40%–60%; light intensity, 150–300 lx; and a 12/12 h light/dark cycle. During the acclimation period, the mice were provided regular meals and ad libitum access to sterilized water. After 1 week of adaptation, the mice were randomly assigned to one of the following seven groups: (I) a control group fed saline (Cont), (II) a DEX group fed saline (DEX), (III) a DEX group fed whey protein isolate (WPI), (IV) a DEX group fed whey protein hydrolysate (WPH), (V) a DEX group fed soy protein isolate (SPI), (VI) a DEX group fed casein (GMP), (VII) a DEX group fed Sal, and (VIII) a DEX group fed F‐Sal (*n* = 8 per group). To induce sarcopenia, 25 mg/kg DEX was administered intraperitoneally every day for 2 weeks to the DEX, WPI, WPH, GMP, SPI, Sal, and F‐Sal groups. At the end of the 2 weeks, the protein sources (WPI, WPH, GMP, ISP, Sal, and F‐Sal) were administered at a dosage equivalent to a daily intake of 500 mg of protein for a 60 kg adult human. During the 2‐week period, the mice were orally administered 105 mg/kg protein samples each day. After the experiment concluded, the mice were sacrificed under ether anesthesia, and their body weights were measured. The quadriceps femoris and gastrocnemius muscles were removed, weighed, and stored at −70°C for gene expression analysis. All research procedures were conducted in accordance with the guidelines set forth by the Institutional Animal Care and Use Committee (IACUC) of Korea University (KU‐IACUC: 2022‐0049).

### 2.6 Microcomputed Tomography (CT) Imaging

The volume and thickness of the gastrocnemius muscles were measured with a high‐resolution micro‐CT scanner (SkyScan 1076; SkyScan, Kontich, Belgium). Additionally, the dissected gastrocnemius muscles from the mice were scanned via a desktop micro‐CT device with a resolution of 5 mm. The scans were performed with a vertical rotation step of 0.6° over 360° (Park et al. 2020). The muscle tissue was identified at a Hounsfield units (HUs) threshold set to 270 ± 100, and the scanned images were reconstructed into 3D images for analysis via CTVox software (version 3.0; SkyScan, Kontich, Belgium) (Park et al. 2020). The cross‐sectional area of each muscle group was analyzed via CTAn software (version 1.18).

### 2.7 Total RNA Isolation and qPCR

The tested cells and mouse tissues subjected to sarcopenic conditions were processed via the RNeasy Mini Kit (Qiagen, Valencia, CA) to isolate total RNA, which was subsequently quantified via a NanoDrop instrument. cDNA was synthesized from the RNA via the PrimeScript 1st Strand cDNA Synthesis Kit (Takara, Tokyo, Japan). To confirm the expression of genes related to sarcopenia from the synthesized cDNA, specific primers for each gene, cDNA, and Prime Q‐Master Mix (GeNet Bio, Nonsan, Korea) were mixed together. Quantitative real‐time PCR (qPCR) was then performed via a CFX96 system (Bio‐Rad, Hercules, CA, US). The mRNA expression levels were normalized to those of the housekeeping gene glyceraldehyde‐3‐phosphate dehydrogenase (GAPDH). The sequences of the primers for gene expression related to sarcopenia are included in the Supporting Information.

### 2.8 Statistical Analysis

Statistical significance was assessed via one‐way analysis of variance (ANOVA) followed by the post hoc Tukey multiple comparison test in GraphPad Prism version 10.0 (GraphPad Software Inc., San Diego, USA). All the statistical analyses were performed in SPSS software, version 22 (IBM Corp., Armonk, NY, USA). All the data are presented as the means ± SDs.

## 3 Results and Discussion

### 3.1 *S. cerevisiae* Significantly Increases the Bound and Free BCAA Contents of Salmon Placenta

Various microbial strains, such as bacteria and fungi, have been reported to play a key role in BCAA production (Hao et al. 2024). And microbial fermentation has been suggested as an effective way to maximize BCAA content (Park and Lee 2010). Therefore, in this study, to validate the effectiveness of salmon placental protein in improving sarcopenia, we first analyzed the amino acid content of salmon placental protein before and after fermentation with *S. cerevisiae* , *A. oryzae* , *L. plantarum* , or *B. subtilis* (Tables 1 and 2). Unfermented Sal contained 118.43 mg of total bound amino acids and 25.99 mg of free amino acids. The samples fermented with the four different strains showed an increasing trend of both bound and free amino acids. Notably, *S. cerevisiae* ‐fermented salmon placental protein demonstrated a significant increase in the bound amino acid content, from 118.43 to 421.22 mg/100 g (more than 3.5‐fold), and the corresponding BCAA content increased by more than 4‐fold, compared with the raw salmon extract. In contrast, Sal fermented with the other strains showed increases of less than two‐fold in bound and free amino acid levels. Additionally, *S. cerevisiae‐*fermented salmon placental protein increased the free amino acid content from 25.99 to 34.44 mg/100 g and the corresponding BCAA content more than 2.5‐fold. The distinction between the free and bound amino acids in this study is because it can be used as a promising tool to assess skeletal muscle health, which can lead to sarcopenia (Calvani et al. 2020). The free amino acids account for only 2% of the total amino acids in the body, but they are quickly absorbed and promote muscle protein synthesis. The bound amino acids are absorbed slowly but provide a continuous supply because they are broken down into free amino acids in the body as protein (Zhenyukh et al. 2017). Therefore, in this study, the considerable increases in the bound and free BCAA amino acid contents induced by *S. cerevisiae* fermentation of salmon placental proteins indicate that it is the most promising strain for producing F‐Sal for managing sarcopenia.

**TABLE 1:** Analysis of the bound amino acid composition and content in 5% aqueous solution (mg/100 g) of raw and fermented salmon placenta proteins.

Amino acid	Salmon placenta	Salmon placental protein fermented with			
		*Saccharomyces cerevisiae*	*Aspergillus oryzae*	*Lactobacillus plantarum*	*Bacillus subtilis*
Leucine	5.99	18.76	10.93	9.74	10.09
Isoleucine	2.47	9.05	5.13	4.11	4.19
Valine	5.06	14.90	9.89	7.71	8.20
Glycine	3.64	50.21	25.43	21.39	22.32
Alanine	6.66	32.66	18.03	15.15	15.32
Serine	20.24	40.43	21.18	18.74	18.85
Proline	—	36.06	11.97	9.69	1.10
Threonine	4.74	15.25	9.10	7.32	7.78
Aspartic acid	20.11	51.03	25.29	19.52	21.81
Lysine	7.56	25.59	13.65	10.84	11.27
Glutamic acid	20.24	62.62	33.04	25.98	27.07
Methionine	2.97	7.33	5.40	4.61	4.88
Histidine	2.76	7.98	4.39	3.93	4.48
Phenylalanine	7.55	10.67	9.74	9.03	8.62
Arginine	6.98	32.92	13.64	13.36	15.53
Tyrosine	1.46	5.76	0.60	1.45	0.85
BCAA (mg/100 g)	13.52	42.71	25.95	21.56	22.48
Total (mg/100 g)	118.43	421.22	217.41	182.57	182.36

**TABLE 2:** Analysis of the free amino acid composition and content in 5% aqueous solution (mg/100 g) of raw and fermented salmon placental proteins.

Amino acid	Salmon placenta	Salmon placental protein fermented with			
		*Saccharomyces cerevisiae*	*Aspergillus oryzae*	*Lactobacillus plantarum*	*Bacillus subtilis*
Leucine	0.93	2.20	1.74	0.73	0.87
Isoleucine	0.65	1.55	1.27	0.23	0.21
Valine	0.81	2.30	1.62	0.46	0.53
Glycine	0.75	1.99	0.82	0.43	0.24
Alanine	1.59	4.32	2.97	1.62	1.39
Serine	2.69	0.25	0.74	0.99	0.16
Proline	0.50	2.89	0.84	1.30	0.40
Threonine	0.77	1.00	0.69	0.16	0.10
Aspartic acid	8.67	2.06	1.44	0.47	0.26
Lysine	1.61	2.30	1.60	0.71	0.16
Glutamic acid	3.66	6.63	3.32	1.02	0.18
Methionine	0.30	0.68	0.56	0.12	0.26
Histidine	0.16	0.48	0.29	0.09	0.04
Phenylalanine	0.48	1.15	0.81	0.17	0.30
Arginine	1.67	2.56	0.13	1.18	0.40
Tyrosine	0.57	1.83	0.71	0.14	0.37
Tryptophan	0.18	0.25	0.27	0.07	0.14
BCAA (mg/100 g)	2.39	6.05	4.63	1.42	1.61
Total (mg/100 g)	25.99	34.44	19.82	9.89	6.01

### 3.2 *F‐Sal* Restores Myotube Morphology and Myogenic Gene Expression While Inhibiting Myoblast Cell Death In Vitro

**FIGURE 1:**
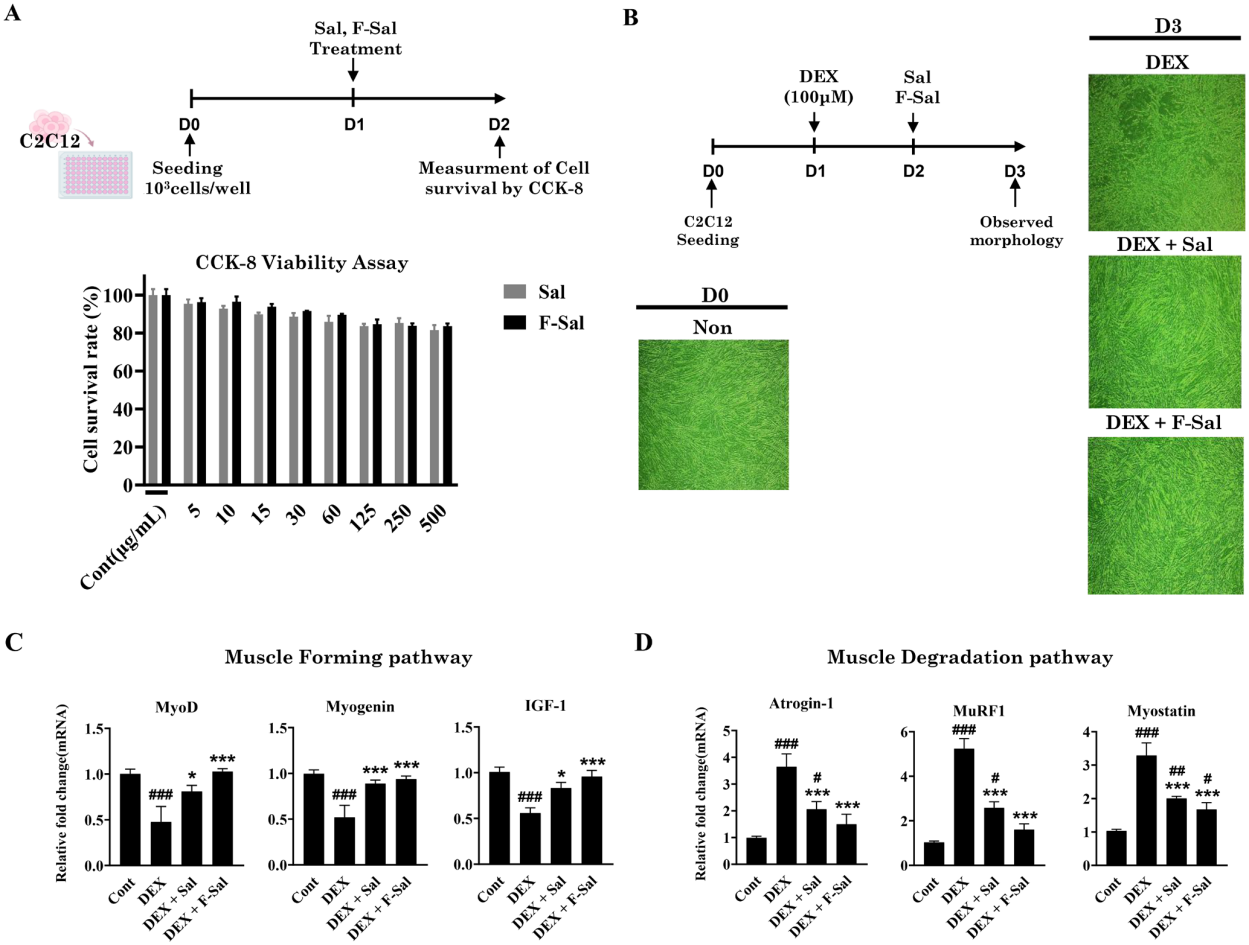
In vitro efficacy of Sal and F‐Sal in a dexamethasone‐induced sarcopenia model in C2C12 cells. (A, B) Schematic representation of the experimental design, showing the timeline of induction of sarcopenic effects, including DEX administration and protein supplementation. (A) Dose‐dependent cytotoxicity and (B) microscopy images following differentiation of C2C12 cells into myotubes (B). Biomarker expression levels related to the prevention (C) and occurrence (D) of sarcopenic effects in C2C12 cells pretreated with Sal and F‐Sal. Bars and error lines represent the means ± SDs; *p < 0.05, **p < 0.01, ***p < 0.001. DEX group, dexamethasone group; F‐Sal, fermented salmon placental proteins; Sal, salmon placental proteins.

F‐Sal has been confirmed as a promising method to improve BCAA content, which is effective in improving sarcopenia, through amino acid analysis experiments. Therefore, we first assessed the dose‐escalating cytotoxic effects of Sal and F‐Sal on C2C12 murine myoblast cells to determine safe doses for treating sarcopenia. One thousand C2C12 cells were seeded onto each well in a 96‐well plate, and cell viability upon treatment with Sal or F‐Sal was assessed using a CCK‐8 assay. The results revealed that even at a concentration of 500 μg/mL, neither Sal nor F‐Sal treatment drastically reduced the viability of C2C12 cells (Figure 1A). However, to ensure that the experiments were performed within a noncytotoxic range of Sal or F‐Sal concentrations, we selected the maximum nontoxic concentration (no observed adverse effect level, NOAEL) of 10 μg/mL, at which the cell survival rate was greater than 90% for the subsequent experiments. Then, we established a model of sarcopenia by treating C2C12 cells with 100 μM DEX 1 day after seeding. On the following day (Day 2), 10 μg/mL Sal or F‐Sal was added to the culture, and its impact on myotube formation was evaluated on Day 3. Compared with the control group, which did not receive DEX or any Sal or F‐Sal, the experimental group treated with DEX alone failed to maintain normal myotube morphology, and the cells exhibited a dispersed and discontinuous appearance. Conversely, the groups treated with DEX + Sal or DEX + F‐Sal retained a morphology similar to that of the control group (Figure 1B). These findings indicate that DEX + Sal and DEX + F‐Sal treatments may help alleviate sarcopenia by preserving a myotube morphology comparable to that in non‐sarcopenic control cells.

To determine what biomarkers are associated with the prevention of sarcopenia in C2C12 cells treated with Sal or F‐Sal, we measured the expression of genes linked to the inhibition of muscle cell death (MyoD, Myogenin), muscle protein synthesis (IGF‐1, insulin‐like growth factor 1), and muscle protein degradation (Atrogin‐1, MuRF1, Myostatin) (Bodine et al. 2001; Gomes et al. 2001; Stevenson et al. 2003). The expression levels of MyoD, Myogenin, and IGF‐1 were significantly lower (*p* < 0.001, *p* < 0.01, *p* < 0.001) in the DEX only‐treated group than in the control group, indicating a loss of muscle‐forming processes (Figure 1C). However, treatment with Sal or F‐Sal restored the expression of all three myogenic genes to levels comparable to those in the control group. Moreover, the addition of Sal or F‐Sal significantly reduced the expression of genes associated with muscle degradation pathways, such as Atrogin‐1, MuRF1, and Myostatin (Figure 1D). Compared with Sal, however, F‐Sal was more effective at increasing the expression of genes involved in muscle formation and in decreasing the expression of those related to muscle degradation pathways. Therefore, the increased expression of MyoD, Myogenin, and IGF‐1 suggests that F‐Sal may increase muscle protein synthesis and overall muscle mass. Furthermore, the increased expression of Atrogin‐1, MuRF1, and Myostatin suggests that F‐Sal may inhibit muscle catabolism and reduce the breakdown of muscle proteins, thus preventing further muscle wasting. Collectively, these data demonstrate that Sal fermented with *S. cerevisiae* could serve as a therapeutic supplement for sarcopenia by modulating key genes and proteins involved in muscle biogenesis, degradation, and cell death.

### 3.3 F‐Sal Improves Sarcopenic Conditions by Increasing the Weight of the Quadriceps and Gastrocnemius Muscles

**FIGURE 2:**
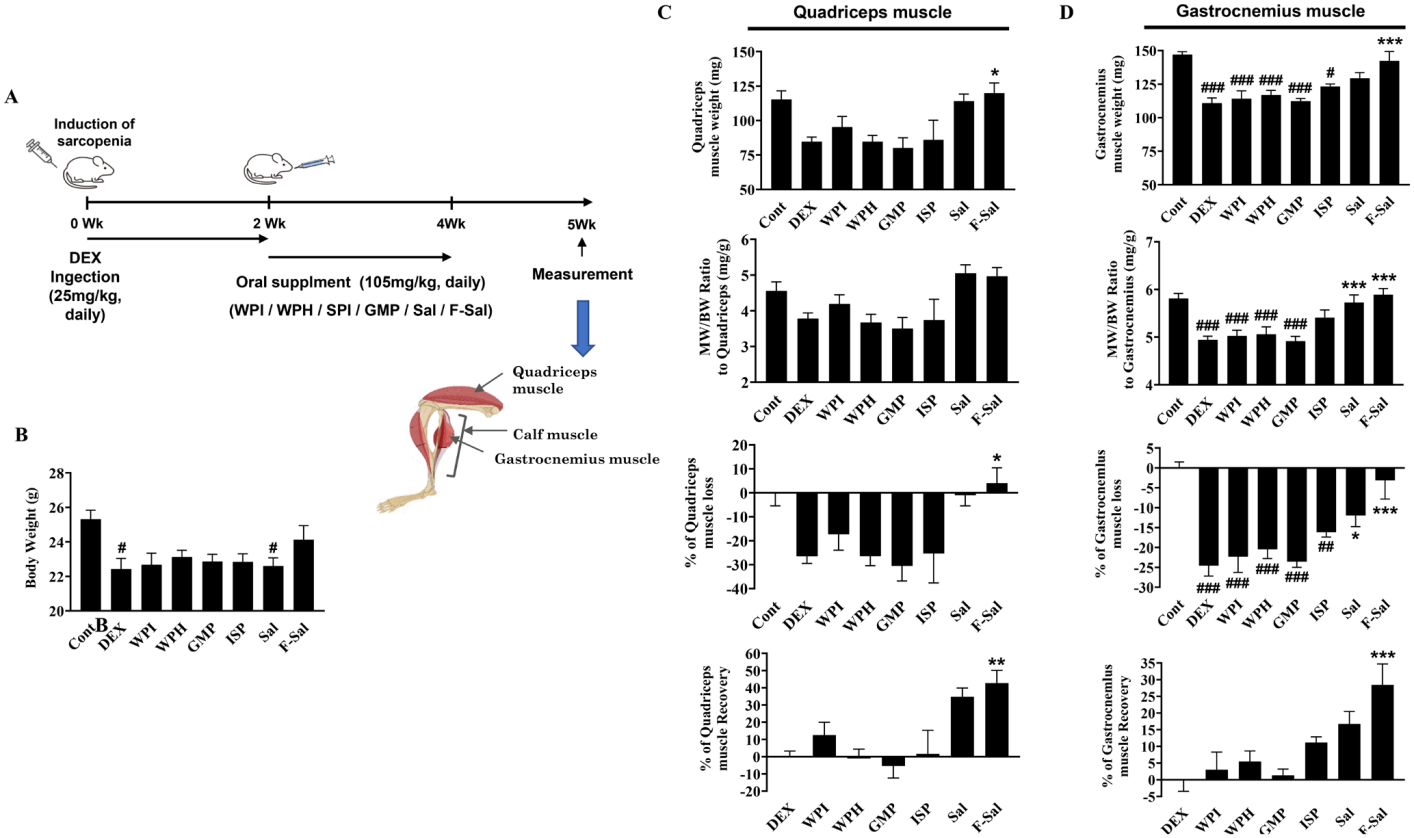
In vivo efficacy of Sal and F‐Sal in a dexamethasone‐induced mouse model of sarcopenia. (A) Schematic representation of the experimental design, showing the timeline of sarcopenia induction, including DEX injection and protein supplementation. (B) Body weights of the mice before and after the administration of Sal, F‐Sal, or other protein supplements. (C) Quadriceps muscle weight, MW/BW ratio, muscle loss, and muscle recovery. (D) Gastrocnemius muscle weight, the MW/BW ratio, muscle loss, and muscle recovery. Bars and error lines represent the means ± SDs. ^#^ *p* < 0.05, ^##^ *p* < 0.01, and ^###^ *p* < 0.001 compared with the control group; *p < 0.05, **p < 0.01, and ***p < 0.001 compared with the DEX group.

Our in vitro data from C2C12 myoblast cells suggested that F‐Sal fermented with *S. cerevisiae* could improve sarcopenic conditions in an animal model of sarcopenia. To test this possibility, sarcopenia was induced in mice by administering DEX at a dose of 25 mg/kg intraperitoneally each day for 2 weeks. Sal or F‐Sal was subsequently administered orally at 105 mg/kg daily for an additional 2 weeks (Figure 2A). As additional comparators, mice were administered whey protein isolate (WPI), whey protein hydrolysate (WPH), casein micellar protein (CMP), or isolated soy protein (ISP).

To assess sarcopenia in terms of muscle loss and recovery, we measured the whole body weight, quadriceps muscle weight, gastrocnemius muscle weight, and the ratio of quadriceps and gastrocnemius muscle weights to total body weight (MW/BW), following the protocols established in previous studies (Park et al. 2020; Engelke et al. 2018; Xie et al. 2021). Compared with control mice, DEX‐treated mice exhibited significant reductions in body weight (*p* < 0.05) (Figure 2B) as well as decreases of 20%–30% in quadriceps and gastrocnemius muscle mass relative to the pretreatment mass (Figure 2C,D). Moreover, DEX‐treated mice receiving the protein supplements (WPI, WPH, CMP, and ISP) experienced similar decreases in muscle mass, indicating that the protein supplement alone does not improve dextromethasone (DEX)‐induced sarcopenia. In contrast, mice treated with Sal or F‐Sal presented muscle weights comparable to those of the control group, increased quadriceps and gastrocnemius muscle weights, and minimal loss, as represented by a 50% recovery from DEX treatment (Figure 2C,D). Additionally, compared with Sal‐treated mice, F‐Sal‐treated mice presented an improved MW/BW ratio and an additional 10%–15% greater muscle mass recovery (Figure 2C,D). Compared with the DEX group, the F‐Sal group showed a 48% increase in muscle recovery, highlighting the effectiveness of this treatment. Overall, these results suggest that F‐Sal fermented with *S. cerevisiae* effectively prevents the loss of quadriceps and gastrocnemius muscle mass in a mouse model of sarcopenia.

### 3.4 F‐Sal Improves Sarcopenic Conditions by Increasing the Gastrocnemius Muscle Volume and Thickness

**FIGURE 3:**
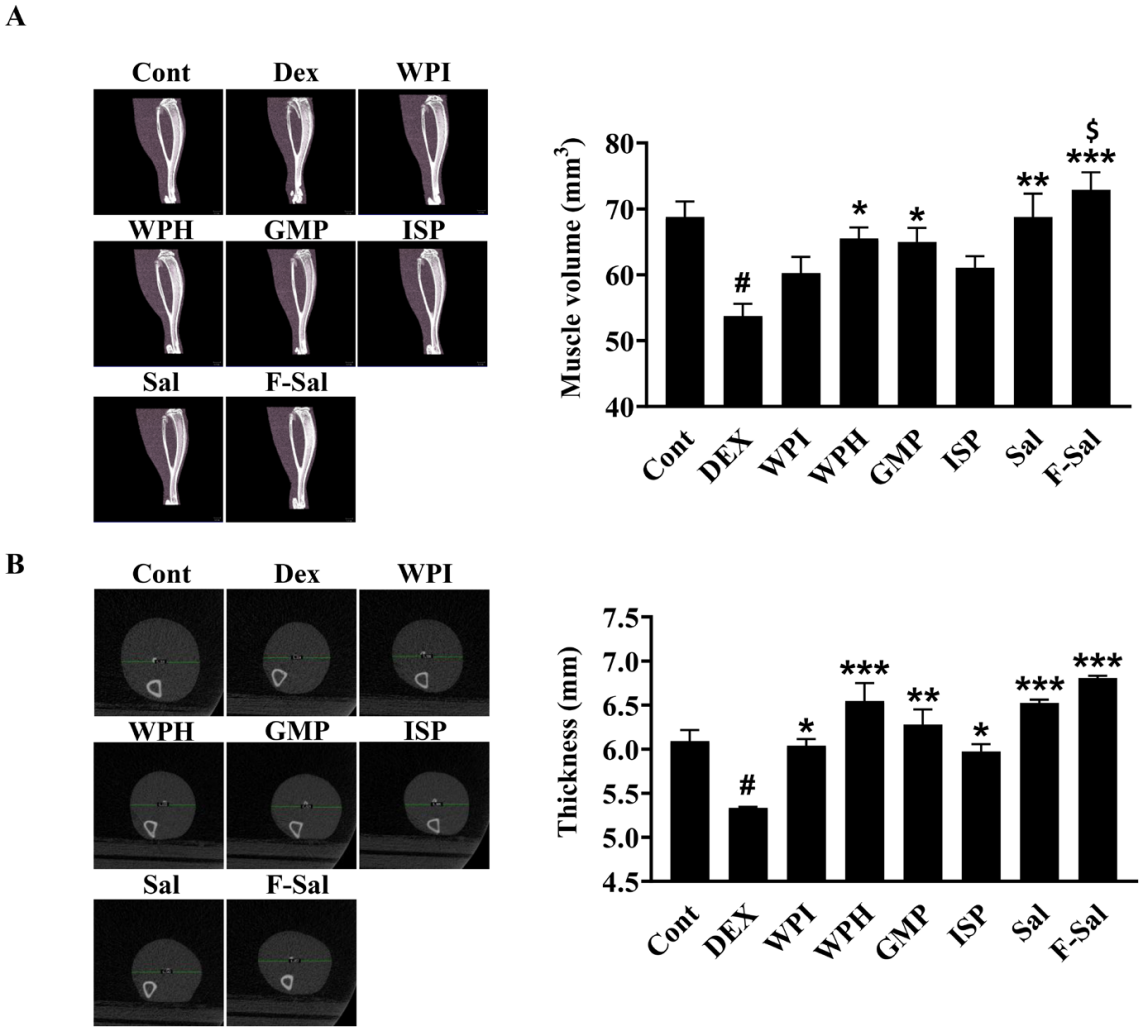
Analysis of cross‐sectional images and volume measurements of the calf muscle in a dexamethasone‐induced mouse model of sarcopenia. Measurement of the cross‐sectional area (A) and thickness (B) of calf muscles in the different experimental groups via micro‐CT imaging. Bars and error lines represent the means ± SDs. ^#^ *p* < 0.05, ^##^ *p* < 0.01, ^###^ *p* < 0.001 compared with the control group; *p < 0.05, **p < 0.01, ***p < 0.001 compared with the DEX group. WPI group, whey protein isolate group.

Given that the increase of the gastrocnemius muscle weight confirmed the improvement of the sarcopenic conditions in a mouse model of sarcopenia, we additionally measured the volume and thickness of the gastrocnemius muscle to further confirm the relationship. Figure 3A shows a sagittal section of the gastrocnemius muscle obtained with micro‐CT, which was used to measure muscle mass by quantitatively analyzing the internal structure of the muscle (Park et al. 2020; Engelke et al. 2018). Compared with the control group, the DEX group exhibited a significant decrease (*p <* 0.05) in calf muscle volume. However, all of the groups administered protein supplements showed a significant increase in calf muscle volume compared with the DEX group. In particular, the F‐Sal experimental group had a greater calf muscle volume (*p <* 0.001) than the other protein‐supplemented groups (WPI, WPH, GMP, and ISP), with volumes comparable to those of the control group (Figure 3A). We also measured the gastrocnemius muscle thickness through cross‐sectional imaging of the gastrocnemius on micro‐CT (Figure 3B) (Park et al. 2020; Engelke et al. 2018). Similar to the gastrocnemius muscle volume, the gastrocnemius muscle thickness was significantly lower (*p <* 0.05) in the DEX group than in the control group and restored in the protein‐supplemented experimental groups, with the greatest gastrocnemius muscle thickness observed in the F‐Sal experimental group (*p* < 0.001) (Figure 3B). These findings indicate that compared with the other protein preparations and the control treatment, F‐Sal fermented with *S. cerevisiae* resulted in greater gastrocnemius muscle mass and thickness, effectively mitigating the muscle loss induced by the sarcopenic conditions induced in the mice. Therefore, based on these research results, it is suggested that F‐Sal can be utilized in the future research and development of health functional foods and therapeutics that improve muscle mass and thickness.

### 3.5 Measurement of Sarcopenia‐Related Biomarker Expression in the Quadriceps Femoris Muscle of the Thigh

**FIGURE 4:**
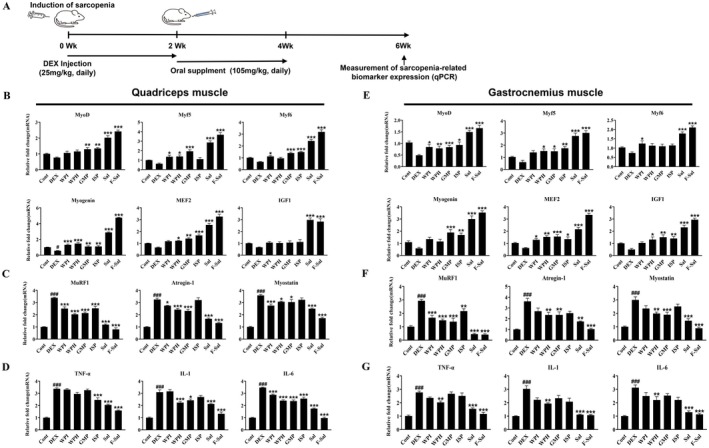
Expression levels of key factors involved in muscle fiber formation, degradation, and inflammation in a dexamethasone‐induced mouse model of sarcopenia. (A) Schematic representation of the experimental design, showing the timeline of sarcopenia induction, including DEX injection, and protein supplementation. (B, E) Expression levels of MyoD, Myf5, Myf6, Myogenin, MEF2, and IGF1 in the quadriceps femoris (B) and gastrocnemius muscles (E). (C, F) Expression levels of MuRF1, Atrogin‐1, and Myostatin in the quadriceps femoris (C) and gastrocnemius muscles (F). (D, G), Expression levels of TNF‐alpha, IL‐1, and IL‐6 in the quadriceps femoris (D) and gastrocnemius muscles (G). Bars and error lines represent the means ± SDs. ^#^ *p* < 0.05, ^##^ *p* < 0.01, and ^###^ *p* < 0.001 compared with the control group; *p < 0.05, **p < 0.01, and ***p < 0.001 compared with the DEX group; ^+^ *p* < 0.05, ^++^ *p* < 0.01, ^+++^ *p* < 0.001 and ^++++^ *p* < 0.0001 compared with the Sal group.

Given that the measurements of gastrocnemius muscle volume and thickness confirmed the effectiveness of the sarcopenia treatment, we sought to further assess the expression of sarcopenia‐related biomarkers in the quadriceps muscle of the thigh. Specifically, we measured the expression levels of MyoD, Myf5, Myf6, Myogenin, and MEF2—key factors involved in muscle fiber formation—and IGF1, a growth factor associated with greater muscle protein synthesis in the quadriceps femoris muscle (McKinnell and Rudnicki 2004; Furrer and Handschin 2019); the corresponding experimental design, including the timeline of sarcopenia induction, DEX injection, protein supplementation, and measurements of biomarker expression, is shown in Figure 4A. MyoD expression was significantly higher in the GMP, ISP, Sal, and F‐Sal groups (*p* < 0.01, *p* < 0.01, *p* < 0.001 and *p* < 0.001) than in the DEX group; similarly, the expression of Myf5 was significantly increased in the WPI, WPH, GMP, Sal, and F‐Sal groups (*p* < 0.05, *p* < 0.05, *p* < 0.001, *p* < 0.001 and *p* < 0.001), and that of Myf6 was significantly increased in the WPI, GMP, ISP, Sal, and F‐Sal groups (*p* < 0.05, *p* < 0.001, *p* < 0.001, *p* < 0.001 and *p* < 0.001) compared to the DEX group. Additionally, all of the protein supplementation groups showed significantly increased Myogenin expression compared to the DEX group, and the WPH, GMP, ISP, Sal, and F‐Sal groups (*p* < 0.05, *p* < 0.01, *p* < 0.001, *p* < 0.001 and *p* < 0.001) also demonstrated significantly higher MEF2 expression levels. Finally, IGF1 expression significantly increased only in the Sal and F‐Sal groups (*p* < 0.001 and *p* < 0.001) compared with that in the DEX group (Figure 4B). These findings indicate that muscle regulatory factors and muscle protein synthesis biomarkers were significantly elevated in the quadriceps femoris muscles of the protein‐treated groups, particularly the F‐Sal group.

In addition, we measured the expression of several factors associated with muscle protein degradation (MuRF1, Atrogin‐1, and Myostatin) and of proinflammatory cytokines that are linked to decreased muscle protein synthesis (TNF‐α, IL‐1, and IL‐6) (McKinnell and Rudnicki 2004; Furrer and Handschin 2019). Compared with the control group, the DEX group presented significantly elevated levels of MuRF1 (*p* < 0.001), Atrogin‐1 (*p* < 0.001), and Myostatin (*p* < 0.001) (Figure 4C), as well as TNF‐α (*p* < 0.001), IL‐1 (*p* < 0.001), and IL‐6 (*p* < 0.001) (Figure 4D). Compared with the DEX group, all the protein supplementation groups presented significantly lower MuRF1 levels (*p* < 0.001), while the WPI, WPH, GMP, Sal, and F‐Sal groups presented significantly reduced Atrogin‐1 (*p* < 0.05, *p* < 0.001, *p* < 0.001, *p* < 0.001, and *p* < 0.001) and Myostatin (*p* < 0.001, *p* < 0.05, *p* < 0.05, *p* < 0.001, and *p* < 0.001) expression. TNF‐α levels were significantly lower in the ISP, Sal, and F‐Sal groups (*p* < 0.001, *p* < 0.001, and *p* < 0.001), whereas IL‐1 levels were significantly lower in the WPH, GMP, Sal, and F‐Sal groups (*p* < 0.001, *p* < 0.05, *p* < 0.001, and *p* < 0.001). IL‐6 expression was significantly lower in all protein supplementation groups than in the DEX group (*p <* 0.001) (Figure 4D).

In summary, compared with the DEX group, the protein supplementation groups presented significantly lower levels of muscle protein degradation and inflammatory biomarkers in the quadriceps femoris muscle. The F‐Sal group, in particular, exhibited reductions in these biomarkers to levels comparable to those of the control group, indicating a substantial improvement. These findings demonstrate that F‐Sal fermented with *S. cerevisiae* effectively increases the expression of myogenic regulators and muscle protein synthesis markers while simultaneously reducing muscle degradation and the expression of inflammatory biomarkers in the quadriceps muscle. In this study, the reduction in the levels of MuRF1 and Atrogin‐1, both of which are components of the ubiquitin–proteasome pathway, suggests that F‐Sal may suppress the proteolytic systems that contribute to muscle loss. Moreover, the expression of Myostatin, a negative regulator of muscle growth, was also significantly reduced in the F‐Sal group. Lowering the expression of Myostatin is crucial because it allows greater muscle growth and regeneration, indicating that F‐Sal promotes a more anabolic muscle environment. Therefore, future research is needed to systematically study the anabolic system of F‐Sal on muscle proteins to establish clinical data that can promote muscle growth and regeneration.

### 3.6 Measurement of Sarcopenia‐Related Biomarker Expression in the Gastrocnemius Muscle

Similar to the analyses performed for the quadriceps muscles, we measured the expression levels of the muscle formation‐related factors MyoD, Myf5, Myf6, Myogenin, and MEF2 and of the muscle protein synthesis‐promoting IGF1 in the gastrocnemius muscle (McKinnell and Rudnicki 2004; Furrer and Handschin 2019). Compared with the DEX group, the protein supplementation groups presented significantly higher MyoD expression. Additionally, Myf5 levels were significantly elevated in the WPH, GMP, ISP, Sal, and F‐Sal groups (*p* < 0.05, *p* < 0.05, *p* < 0.01, *p* < 0.001, and *p* < 0.001). Moreover, compared with the DEX group, the WPI, Sal, and F‐Sal groups demonstrated significantly greater Myf6 expression levels (*p* < 0.05, *p* < 0.001, and *p* < 0.001), whereas the GMP, ISP, Sal, and F‐Sal groups showed notably higher levels of Myogenin expression (*p* < 0.001, *p* < 0.01, *p* < 0.001, and *p* < 0.001). Finally, compared with those in the DEX group, MEF2 levels were significantly increased across all protein supplementation groups, while IGF1 expression was significantly increased in the WPH, GMP, ISP, Sal, and F‐Sal groups (*p* < 0.05, *p* < 0.01, *p* < 0.01, *p* < 0.001, and *p* < 0.001) (Figure 4E). Thus, the protein supplementation groups showed significant increases in muscle regulatory factors and biomarkers associated with muscle protein synthesis, with F‐Sal showing the greatest increases.

We also measured the expression levels of the muscle protein degradation‐related factors MuRF1, Atrogin‐1, and Myostatin and the proinflammatory (and anti‐muscle protein synthesis) cytokines TNF‐α, IL‐1, and IL‐6 (McKinnell and Rudnicki 2004; Furrer and Handschin 2019). Compared with the control group, the DEX group presented significant increases (*p* < 0.001) in MuRF1, Atrogin‐1, Myostatin, TNF‐α, IL‐1, and IL‐6 levels (Figure 4F,G). However, compared with those in the DEX group, MuRF1 levels in all protein supplementation groups were significantly lower, whereas Atrogin‐1 and Myostatin levels were significantly lower in the WPH, GMP, Sal, and F‐Sal groups. Furthermore, TNF‐α, IL‐1, and IL‐6 levels were significantly reduced in the WPH, Sal, and F‐Sal groups (Figure 4).

In summary, the protein supplementation groups presented significantly lower biomarkers related to muscle protein degradation and decreased protein synthesis than the DEX group did in the gastrocnemius muscle. In particular, F‐Sal treatment resulted in the greatest reduction, resulting in levels close to those of the control group. These results indicate that Sal (Salmon Placental Protein) fermented with *S. cerevisiae* effectively increases the expression of Myogenic regulators and muscle protein synthesis while simultaneously reducing the expression of markers of muscle protein degradation and inflammation in the gastrocnemius muscle. In this study, F‐Sal protein supplementation significantly decreased the expression of these cytokines (TNF‐α, IL‐1, and IL‐6), indicating that the added proteins induced anti‐inflammatory effects. By reducing inflammation, F‐Sal may help restore the balance between muscle synthesis and degradation, thus supporting muscle preservation and regeneration. The anti‐inflammatory properties of F‐Sal might be partly attributed to the bioactive compounds generated during fermentation by *S. cerevisiae* , which have been shown to have immunomodulatory effects. This reduction in systemic inflammation could play an important role in mitigating sarcopenia and promoting healthier muscle aging. Therefore, future research is needed to identify specific bioactive compounds in F‐Sal and to systematically investigate the anti‐inflammatory effects of F‐Sal.

## 4 Conclusions

Salmon placental protein fermented with *S. cerevisiae* (F‐Sal) is a promising method to improve BCAA content, which is effective in inhibiting muscle loss. The BCAA content of free amino acid of F‐Sal significantly increased from 2.39 to 6.05 mg/100 g (more than 2.5‐fold) compared to raw salmon placental protein, and the BCAA content of bound amino acid of F‐Sal significantly increased from 13.52 to 42.71 mg/100 g (more than three‐fold) compared to raw salmon placental protein. F‐Sal with enhanced BCAA content upregulated muscle building genes and downregulated the expression of muscle breakdown markers more than other protein supplements. Additionally, its ability to lower the levels of proinflammatory cytokines such as TNF‐α, IL‐1, and IL‐6 further highlights its therapeutic potential for reducing muscle inflammation and promoting muscle health. Overall, the findings of this study suggest that F‐Sal fermented with *S. cerevisiae* is a highly effective intervention for sarcopenia that outperforms other protein supplements in terms of indicators of the disease such as muscle mass and the expression of muscle formation‐related factors in a mouse model of sarcopenia. Therefore, F‐Sal can provide a therapeutic strategy that is highly beneficial for the management of sarcopenia, particularly in aging populations. Future studies should accumulate long‐term clinical data on F‐Sal for sarcopenia and explore its effects on muscle‐related diseases such as myopathy, neuromuscular disease, and musculoskeletal disease.

## Author Contributions

**Jung‐Chang Lee:** conceptualization (equal), investigation (equal), methodology (equal), project administration (equal), supervision (equal), validation (equal), writing – original draft (equal), writing – review and editing (equal). **Tae‐Joong Lim:** conceptualization (equal), formal analysis (equal), methodology (equal), writing – review and editing (equal). **Hye‐Min Lee:** data curation (equal), formal analysis (equal), software (equal), visualization (equal), writing – review and editing (equal). **Sung‐Moon Jun:** data curation (equal), investigation (equal), methodology (equal), resources (equal), software (equal). **Kyung‐Mi Lee:** conceptualization (equal), funding acquisition (equal), project administration (equal), supervision (equal), validation (equal), writing – review and editing (equal).

## Disclosure

Declaration of generative AI and AI‐assisted technologies: We confirm that no artificial intelligence tools were used in the preparation of this manuscript.

## Ethics Statement

The animal testing plan included in this study was implemented after the ethical and scientific nature of the animal testing therein was reviewed by the Institutional Animal Care and Use Committee (IACUC) of Korea University, and approval was obtained as appropriate (KU‐IACUC: 2022‐0049).

## Conflicts of Interest

The authors declare no conflicts of interest.

## Supporting information


**Data S1:** fsn371370‐sup‐0001‐Supinfo.docx.

## Data Availability

The data that support the findings of this study are available from the corresponding author upon reasonable request.

## References

[fsn371370-bib-0001] Ahmmed, M. K. , A. Carne , F. Ahmmed , I. Stewart , H. (Sabrina) Tian , and A. E. D. A. Bekhit . 2021. “Positional Distribution of Fatty Acids and Phospholipid Composition in King Salmon ( *Oncorhynchus tshawytscha* ) Head, Roe and Skin Using Nuclear Magnetic Resonance Spectroscopy.” Food Chemistry 363: 130302. 10.1016/j.foodchem.2021.130302. 34130099

[fsn371370-bib-0002] Arnarson, A. , O. G. Geirsdottir , A. Ramel , et al. 2013. “Effects of Whey Proteins and Carbohydrates on the Efficacy of Resistance Training in Elderly People: Double Blind, Randomised Controlled Trial.” European Journal of Clinical Nutrition 67: 821–826. 10.1038/ejcn.2013.40. 23486511

[fsn371370-bib-0003] Banerjee, K. K. , A. Bishayee , and M. Chatterjee . 1992. “Anti‐Inflammatory Effect of Human Placental Extract: A Biochemical Mechanistic Approach.” Rivista Europea per le Scienze Mediche e Farmacologiche 14: 361–366. 1308603

[fsn371370-bib-0004] Bauer, J. M. , S. Verlaan , I. Bautmans , et al. 2015. “Effects of a Vitamin D and Leucine‐Enriched Whey Protein Nutritional Supplement on Measures of Sarcopenia in Older Adults, the PROVIDE Study: A Randomized, Double‐Blind, Placebo‐Controlled Trial.” Journal of the American Medical Directors Association 16: 740–747. 10.1016/j.jamda.2015.05.021. 26170041

[fsn371370-bib-0005] Beeri, M. S. , S. E. Leugrans , O. Delbono , D. A. Bennett , and A. S. Buchman . 2021. “Sarcopenia Is Associated With Incident Alzheimer's Dementia, m Ild Cognitive Impairment, and Cognitive Decline.” Journal of the American Geriatrics Society 69, no. 7: 1826–1835. 10.1111/jgs.17206. 33954985 PMC8286176

[fsn371370-bib-0006] Bekhit, A. E. D. A. , A. Duncan , C. S. F. Bah , I. A. M. Ahmed , F. Y. al‐Juhaimi , and H. F. Amin . 2018. “Impact of Fermentation Conditions on the Physicochemical Properties, Fatty Acid and Cholesterol Contents in Salted‐Fermented Hoki Roe.” Food Chemistry 264: 73–80. 10.1016/j.foodchem.2018.05.008. 29853407

[fsn371370-bib-0007] Bekhit, A. E. D. A. , J. D. Morton , C. O. Dawson , J. H. Zhao , and H. Y. Y. Lee . 2009. “Impact of Maturity on the Physicochemical and Biochemical Properties of Chinook Salmon Roe.” Food Chemistry 117: 318–325. 10.1016/j.foodchem.2009.04.009.

[fsn371370-bib-0008] Bodine, S. C. , E. Latres , S. Baumhueter , et al. 2001. “Identification of Ubiquitin Ligases Required for Skeletal Muscle Atrophy.” Science 294: 1704–1708. 10.1126/science.1065874. 11679633

[fsn371370-bib-0009] Bunga, S. J. , M. K. Ahmmed , B. Lawley , A. Carne , and A. E. D. A. Bekhit . 2023. “Physicochemical, Biochemical and Microbiological Changes of Jeotgal‐Like Fermented Chinook Salmon ( *Oncorhynchus tshawytscha* ) roe.” Food Chemistry 398: 133880. 10.1016/j.foodchem.2022.133880. 35986997

[fsn371370-bib-0010] Calvani, R. , L. Rodriguez‐Mañas , A. Picca , et al. 2020. “Identification of a Circulating Amino Acid Signature in Frail Older Persons With Type 2 Diabetes Mellitus: Results From the Metabofrail Study.” Nutrients 12: 199. 10.3390/nu12010199. 31940925 PMC7019630

[fsn371370-bib-0011] Carducci, C. , M. Birarelli , V. Leuzzi , G. Santagata , P. Serafini , and I. Antonozzi . 1996. “Automated Method for the Measurement of Amino Acids in Urine by High‐Performance Liquid Chromatography.” Journal of Chromatography. A 729: 173–180. 10.1016/0021-9673(95)00964-7. 9004938

[fsn371370-bib-0012] Engelke, K. , O. Museyko , L. Wang , and J.‐D. Laredo . 2018. “Quantitative Analysis of Skeletal Muscle by Computed Tomography Imaging‐State of the Art.” Journal of Orthopaedic Translation 15: 91–103. 10.1016/j.jot.2018.10.004. 30533385 PMC6260391

[fsn371370-bib-0013] Furrer, R. , and C. Handschin . 2019. “Muscle Wasting Diseases: Novel Targets and Treatments.” Annual Review of Pharmacology and Toxicology 59: 315–339. 10.1146/annurev-pharmtox-010818-021041. PMC6701981 30148697

[fsn371370-bib-0014] Gomes, M. D. , S. H. Lecker , R. T. Jagoe , A. Navon , and A. L. Goldberg . 2001. “Atrogin‐1, a Muscle‐Specific F‐Box Protein Highly Expressed During Muscle Atrophy.” Proceedings of the National Academy of Sciences of the United States of America 98: 14440–14445. 10.1073/pnas.251541198. 11717410 PMC64700

[fsn371370-bib-0015] Hao, Y. , X. Pan , J. You , G. Li , M. Xu , and Z. Rao . 2024. “Microbial Production of Branched Chain Amino Acids: Advances and Perspectives.” Bioresource Technology 397: 130502. 10.1016/j.biortech.2024.130502. 38417463

[fsn371370-bib-0016] Kim, Y. W. , S. R. Baek , E. S. Lee , et al. 2015. “Wound Healing Effects of Rose Placenta in a Mouse Model of Full‐Thickness Wounds.” Archives of Plastic Surgery 42: 686–694. 10.5999/aps.2015.42.6.686. 26618114 PMC4659980

[fsn371370-bib-0017] Kwak, J. Y. , and K. S. Kwon . 2019. “Pharmacological Interventions for Treatment of Sarcopenia: Current Status of Drug Development for Sarcopenia.” Annals of Geriatric Medicine and Research 23: 98–104. 10.4235/agmr.19.0028. 32743297 PMC7370765

[fsn371370-bib-0018] Lee, K. H. , T. H. Kim , W. C. Lee , S. H. Kim , S. Y. Lee , and S. M. Lee . 2011. “Anti‐Inflammatory and Analgesic Effects of Human Placenta Extract.” Natural Product Research 25: 1090–1100. 10.1080/14786419.2010.489050. 21726131

[fsn371370-bib-0019] Ljungdahl, P. O. , and B. Daignan‐Fornier . 2012. “Regulation of Amino Acid, Nucleotide, and Phosphate Metabolism in *Saccharomyces cerevisiae* .” Genetics 190: 885–929. 10.1534/genetics.111.133306. 22419079 PMC3296254

[fsn371370-bib-0020] McKinnell, I. W. , and M. A. Rudnicki . 2004. “Molecular Mechanisms of Muscle Atrophy.” Cell 119: 907–910. 10.1016/j.cell.2004.12.007. 15620349

[fsn371370-bib-0021] Moriya, H. , T. Kuniminato , M. Hosokawa , K. Fukunaga , T. Nishiyama , and K. Miyashita . 2007. “Oxidative Stability of Salmon and Herring Roe Lipids and Their Dietary Effect on Plasma Cholesterol Levels of Rats.” Fisheries Science 73: 668–674. 10.1111/j.1444-2906.2007.01380.x.

[fsn371370-bib-0022] Morley, J. E. 2016. “Pharmacologic Options for the Treatment of Sarcopenia.” Calcified Tissue International 98: 319–333. 10.1007/s00223-015-0022-5. 26100650

[fsn371370-bib-0023] Naseeb, M. A. , and S. L. Volpe . 2017. “Protein and Exercise in the Prevention of Sarcopenia and Aging.” Nutrition Research 40: 1–20. 10.1016/j.nutres.2017.01.001. 28473056

[fsn371370-bib-0024] Pan, S. Y. , M. Chan , M. Wong , D. Klokol , and V. Chernykh . 2017. “Placental Therapy: An Insight to Their Biological and Therapeutic Properties.” Journal of Medical Therapeutics 1: 1–6. 10.15761/JMT.1000118.

[fsn371370-bib-0025] Park, J. H. , and S. Y. Lee . 2010. “Fermentative Production of Branched Chain Amino Acids: A Focus on Metabolic Engineering.” Applied Microbiology and Biotechnology 85: 491–506. 10.1007/s00253-009-2307-y. 19844702

[fsn371370-bib-0026] Park, S. H. , J. Oh , M. Jo , et al. 2020. “Water Extract of Lotus Leaf Alleviates Dexamethasone‐Induced Muscle Atrophy via Regulating Protein Metabolism‐Related Pathways in Mice.” Molecules (Basel, Switzerland) 25: 4592. 10.3390/molecules25204592. 33050143 PMC7587191

[fsn371370-bib-0027] Park, S. Y. , S. Phark , M. Lee , J. Y. Lim , and D. Sul . 2010. “Anti‐Oxidative and Anti‐Inflammatory Activities of Placental Extracts in Benzo[a]Pyrene‐Exposed Rats.” Placenta 31: 873–879. 10.1016/j.placenta.2010.07.010. 20708262

[fsn371370-bib-0028] Philipps, A. F. , I. R. Holzman , C. Teng , and F. C. Battaglia . 1978. “Tissue Concentrations of Free Amino Acids in Term Human Placentas.” American Journal of Obstetrics and Gynecology 131: 881–887. 10.1016/S0002-9378(16)33136-2. 686088

[fsn371370-bib-0029] Quan, Y. , C. Wang , L. Wang , and G. Li . 2023. “Geriatric Sarcopenia Is Associated With Hypertension: A Systematic Review and Meta‐Analysis.” Journal of Clinical Hypertension (Greenwich, Conn.) 25, no. 9: 808–816. 10.1111/jch.14714. 37594142 PMC10497027

[fsn371370-bib-0030] Sandri, M. , C. Sandri , A. Gilbert , et al. 2004. “Foxo Transcription Factors Induce the Atrophy‐Related Ubiquitin Ligase Atrogin‐1 and Cause Skeletal Muscle Atrophy.” Cell 117: 399–412. 10.1016/s0092-8674(04)00400-3. 15109499 PMC3619734

[fsn371370-bib-0031] Stevenson, E. J. , P. G. Giresi , A. Koncarevic , and S. C. Kandarian . 2003. “Global Analysis of Gene Expression Patterns During Disuse Atrophy in Rat Skeletal Muscle.” Journal of Physiology 551: 33–48. 10.1111/j.1469-7793.2003.00033.x. 12844509 PMC2343139

[fsn371370-bib-0032] Sur, T. K. , T. K. Biswas , L. Ali , et al. 2013. “Anti‐Cancer Activity of *Glycosmis parva* Leaf Extract on Human Colorectal Cancer HT29 Cell.” Acta Pharmacologica Sinica 24: 187–192. 12546729

[fsn371370-bib-0033] ter Borg, S. , Y. C. Luiking , A. van Helvoort , Y. Boirie , J. M. G. A. Schols , and C. P. G. M. de Groot . 2019. “Low Levels of Branched Chain Amino Acids, Eicosapentaenoic Acid and Micronutrients Are Associated With Low Muscle Mass, Strength and Function in Community‐Dwelling Older Adults.” Journal of Nutrition, Health & Aging 23: 27–34. 10.1007/s12603-018-1108-3. PMC12280390 30569065

[fsn371370-bib-0034] Togashi, S. I. , N. Takahashi , Y. Kubo , et al. 2000. “Purification and Identification of Antioxidant Substances in Human‐Placenta Extracts.” Journal of Health Science 46: 117–125. 10.1248/jhs.46.117.

[fsn371370-bib-0035] Volpi, E. , H. Kobayashi , M. Sheffield‐Moore , B. Mittendorfer , and R. Wolfe . 2003. “Essential Amino Acids Are Primarily Responsible for the Amino Acid Stimulation of Muscle Protein Anabolism in Healthy Elderly Adults.” American Journal of Clinical Nutrition 78: 250–258. 10.1093/ajcn/78.2.250. 12885705 PMC3192452

[fsn371370-bib-0036] Xie, W. Q. , M. He , D. J. Yu , et al. 2021. “Mouse Models of Sarcopenia: Classification and Evaluation.” Journal of Cachexia, Sarcopenia and Muscle 12: 538–554. 10.1002/jcsm.12709. 33951340 PMC8200444

[fsn371370-bib-0037] Yoshimura, Y. , K. Uchida , S. Jeong , and M. Yamaga . 2016. “Effects of Nutritional Supplements on Muscle Mass and Activities of Daily Living in Elderly Rehabilitation Patients With Decreased Muscle Mass: A Randomized Controlled Trial.” Journal of Nutrition, Health & Aging 20: 185–191. 10.1007/s12603-015-0570-4.26812515

[fsn371370-bib-0038] Zhenyukh, O. , E. Civantos , M. Ruiz‐Ortega , et al. 2017. “High Concentration of Branched‐Chain Amino Acids Promotes Oxidative Stress, Inflammation and Migration of Human Peripheral Blood Mononuclear Cells via mTORC1 Activation.” Free Radical Biology & Medicine 104: 165–177. 10.1016/j.freeradbiomed.2017.01.009. 28089725

[fsn371370-bib-0039] Zhou, Y. , M. Yang , J. Yin , et al. 2023. “Physicochemical Characteristics and Gel‐Forming Properties of Mandarin Fish ( *Siniperca chuatsi* ) Protein During the Fish Fermentation With *Lactobacillus Sake* SMF‐L5: The Formation of Garlic‐Cloves Shaped Protein Gel.” Food Chemistry 409: 135282. 10.1016/j.foodchem.2022.135282. 36577324

