# Supplementary material for: Saccharomyces cerevisiae‐Fermented Salmon Placental Protein Inhibits Muscle Loss in a Mouse Model of Sarcopenia
Source: Food Sci Nutr. 2025 Dec 29;14(1):e71370. doi: 10.1002/fsn3.71370 (PMC12745894; doi:10.1002/fsn3.71370)
Supplement: Supplementary file 1 — Data S1: fsn371370‐sup‐0001‐Supinfo.docx. [file FSN3-14-e71370-s001.docx]

**Supplementary Material 1**

**1. Protein identification for F-Sal and Sal**

***1.1. Materials***

**Solvents:**

Water (Burdick & Jackson, AH365-4), acetonitrile (Burdick & Jackson, AH015-4), and formic acid (Sigma, 64-18-6)

**Reagents:**

The following reagents were used in the study: ammonium bicarbonate (ABC) (Sigma, A6141), iodoacetamide (Sigma, I1149), DL-dithiothreitol (DTT) (Sigma, D0632), urea (Sigma, U0631), Tris-HCl (Bio-Rad, 161-0798), anhydrous calcium chloride (Sigma, C4901), and trypsin (Roche, 11418025001). All reagents were of analytic quality or greater.

***1.2 In-gel protein tryptic digestion***

Protein samples were separated using SDS‒polyacrylamide gel electrophoresis (SDS‒PAGE). The gel was then separately destained with water, 0.1 M ammonium bicarbonate (ABC), 0.1 M ABC/acetonitrile solution (1:1, v/v) and acetonitrile under shaking at 800 rpm for 15 min each. The remaining solution was discarded, and the process was repeated twice. The destained gel was then dried by speed vacuuming to eliminate the remaining solution from the gel. The proteins were reduced by the addition of 0.5 mL of 100 mM dithiothreitol (DTT) in 100 mM ABC, followed by incubation at 37°C for 1 hr. Alkylation was performed by adding 0.5 mL of 55 mM iodoacetamide (IAA) in 100 mM ABC at room temperature for 30 min in the dark. For trypsin digestion, trypsin (1:50, v/v) was added to the protein solution, and the mixture was incubated at 37°C for 18 hr. When digestion was complete, 0.5 mL of 5% formic acid/acetonitrile (2:1, v/v) was added. The digested sample was subsequently concentrated by speed vacuuming. The final sample was prepared for liquid chromatography–mass spectrometry (LC‒MS) analysis after solvent evaporation with the addition of 13 µL of water.

***1.3 Liquid chromatography–mass spectrometry (LC‒MS) analysis***

**Chromatography:**

Ultrapure LC–MS (UPLC‒MS) analysis of the digestion products was performed using a TripleTOF 5600+ (ABSCIEX) coupled to a 1290 Infinity II (Agilent). A Waters Acquity UPLC^®^ C18 column (1.7 µm, 2.1×150 mm) was used with a column temperature of 40°C and a flow rate of 300 µL/min; 13 µl of the digested, reduced and alkylated sample was injected onto the column. Mobile phase A consisted of 0.1% formic acid in water, while mobile phase B consisted of 0.1% formic acid in acetonitrile. The gradient started at 2% B for 5 min and then increased gradually to 45% after 120 min. Next, the gradient was increased from 45% B to 100% B within 5 min. Finally, the column was washed with 100% B for 5 min, followed by equilibration at 2% B for 5 min.

**Mass spectrometry:**

Acquisition was performed on a quadrupole time-of-flight mass spectrometer over a full scan from m/z 50 to 3,000. The electrospray parameters were as follows: spray voltage 5,500 V, curtain gas pressure 25 psi, ion source gas 1 and gas 2 pressure 50 psi, and ion source temperature 500°C. The MS/MS scan method was applied using a collision energy (CE) and a declustering potential (DP) of 3 and 80 V, respectively.

***1.4 Data analysis***

The mass spectrometry data were searched against the data from *Oncorhynchus keta* using ProteinPilot^TM^ software (ABSCIEX) and PeakView^®^ software (ABSCIEX). For the ProteinPilot^TM^ search, the total intensity threshold was set to 0.05% of the base chromatogram peak top intensity.

**2. Total RNA isolation and qPCR for sarcopenia gene expression**

The gene expression levels of the tested cells and mouse tissues under sarcopenic conditions were measured. The sequences of the primers used for qPCR were as follows: MyoD, 5'-GCC GGT GTG CAT TCC AA-3' (forward) and 5' CAC TCC GGA ACC CCA ACA G-3' (reverse);

Myf5, 5'-CCA CCT CCA ACT GCT CTG AT-3' (forward) and 5'-TGA AGC CTT CTT CGT CCT GT-3' (reverse); Myf6, 5'-CTT GAG GGT GCG GAT TTC CT-3' (forward) and 5'-TCC ACG TTT GCT CCT CCT TC-3' (reverse); myogenin, 5'-AGC ATC ACG GTG GAG GAT ATG-3' (forward), and 5'-CAG TTG GGC ATG GTT TCG T-3' (reverse); myocyte enhancer factor 2 (MEF2), 5'-TCC ATC AGC CAT TTC AAC AA-3' (forward) and 5'-GTT ACA GAG CCG AGG TGG AG-3' (reverse); insulin-like growth factor-1 (IGF1), 5'-GCT GGT GGA TGC TCT TCA GT-3' (forward) and 5'-TCC GGA AGC AAC ACT CAT CC-3' (reverse); MuRF1, 5'-TGT CTG GAG GTC GTT TCC G-3' (forward) and 5 '-TGC CGG TCC ATG ATC ACT T-3' (reverse); atrogin-1, 5'-CTC TGT ACC ATG CCG TTC CT-3' (forward) and 5'-GGC TGC TGA ACA GAT TCT CC-3' (reverse); myostatin, 5'-TGT CTG GAG GTC GTT TCC G-3' (forward) and 5'-TGC CGG TCC ATG ATC ACT T-3' (reverse); TNF-α, 5'- ATG GCC TCC CTC TCA TCA GT-3' (forward) and 5'-CAC TTG GTG GTT TGC TAC GA-3' (reverse); IL-1, 5'-GAA GCT CGT CAG GCA GAA GT-3' (forward) and 5'-GTG CAC CCG ACT TTG TTC TT-3' (reverse); IL-6, 5'-CAT AGC TAC CTG GAG TAC ATG A-3' (forward) and 5'-CAT TCA TAT TGT CAG TTC TTC G-3' (reverse); and GAPDH, 5'-CAT GGC CTT CCG TGT TCC TA-3' (forward) and 5'-GCG GCA CGT CAG ATC CA-3' (reverse).

**Supplementary Material 2**

1. **Material & Method**
   1. **Materials**
2. Solvent DW, ACN (B&J)
3. Reagent

Formic acid (Sigma-Aldrich)

Urea (Sigma-Aldrich)

Iodoacetamide (Sigma-Aldrich)

Trypsin (Roche)

Tris-HCl (Bio-Rad)

- 1. **Trypsin digestion**

1. Reduction 6M Urea and 200mM DTT in 50mM Tris-HCl
2. Alkylation 200mM IAA in 50mM Tris-HCl
3. Digestion 100ng/μL Trypsin, 18hr incubation at 37oC
4. Stop reaction Formic acid
5. **Instrument Condition**
   1. **LC method**
6. Chromatography Agilent 1280
7. Mass spectrometry AB SCIEX Q-TOF 5600+
8. Column Acquity UPLC® C18, 1.7um , 2.1 x 150 mm

4. Solvent Solvent

DW (0.1% FOA): ACN (0.1% FOA)

| Time | Flow rate (mL/min) | A(%) | B(%) |
| --- | --- | --- | --- |
| 0.0 | 0.3 | 98 | 2 |
| 5.0 | 0.3 | 98 | 2 |
| 120.0 | 0.3 | 55 | 45 |
| 125.0 | 0.3 | 0 | 100 |
| 130.0 | 0.3 | 0 | 100 |
| 131.0 | 0.3 | 98 | 2 |
| 135.0 | 0.3 | 98 | 2 |

5. Flow rate

6. Injection vol. vol.

13μL uL

- 1. **MS method**

1. Detection ion mode Positive([M+H]+)
2. Mass spectrometry MS: *m/z* 50~2500
3. Spray 3.5 kV
4. Capillary voltage 20V
5. Capillary Temp. 350 ℃
6. Software Peak view, Protein pilot
7. **Results**
   1. **F-Sal (*Saccharomyces cerevisiae*-fermented salmon placental protein)**

**3.1.2. Protein ID lists**

| **N** | **Name** | **Species** | **Peptides**  **(95%)** | **%**  **Coverage** |
| --- | --- | --- | --- | --- |
| 1 | Vitellogenin [*Oncorhynchus keta*] | *Oncorhynchus keta* | 25 | 18.6 |
| 2 | Keratin, type I cytoskeletal 13-like [*Oncorhynchus keta*] | *Oncorhynchus keta* | 1 | 10.7 |
| 3 | Keratin, type II cytoskeletal 8 [*Oncorhynchus keta*] | *Oncorhynchus keta* | 4 | 8.2 |
| 4 | Intermediate filament protein ON3-like [*Oncorhynchus keta*] | *Oncorhynchus keta* | 4 | 7.3 |
| 5 | Elongation factor 1-alpha, somatic form-like [*Oncorhynchus keta*] | *Oncorhynchus keta* | 1 | 6.9 |
| 6 | Keratin, type I cytoskeletal 50 kDa-like [*Oncorhynchus keta*] | *Oncorhynchus keta* | 1 | 6.2 |
| 7 | Keratin, type I cytoskeletal 50 kDa-like [*Oncorhynchus keta*] | *Oncorhynchus keta* | 1 | 3.8 |
| 8 | REVERSED phospholipid-transporting ATPase ID-like [*Oncorhynchus keta*] | *Oncorhynchus keta* | 0 | 1.2 |
| 9 | Axonemal dynein light chain domain-containing protein 1 isoform X1 [*Oncorhynchus keta*] | *Oncorhynchus keta* | 0 | 0.7 |
| 10 | Microtubule-associated protein 2-like isoform X18 [*Oncorhynchus keta*] | *Oncorhynchus keta* | 1 | 0.6 |

- 1. **Sal (Salmon placental protein)**

**3.2.1. Protein ID lists**

|  | **Name** | **Species** | **Peptides**  **(95%)** | **%**  **Coverage** |
| --- | --- | --- | --- | --- |
| 1 | Vitellogenin, partial [*Oncorhynchus keta*] | *Oncorhynchus keta* | 41 | 67.9 |
| 2 | Vitellogenin, partial [*Oncorhynchus keta*] | *Oncorhynchus keta* | 38 | 61.7 |
| 3 | Vitellogenin [*Oncorhynchus keta*] | *Oncorhynchus keta* | 121 | 46.8 |
| 4 | Vitellogenin, partial [*Oncorhynchus keta*] | *Oncorhynchus keta* | 13 | 45.4 |
| 5 | Hemoglobin subunit beta-1 [*Oncorhynchus keta*] | *Oncorhynchus keta* | 3 | 21.8 |
| 6 | Hemoglobin subunit beta [*Oncorhynchus keta*] | *Oncorhynchus keta* | 3 | 18.2 |
| 7 | Trypsin-3 [*Oncorhynchus keta*] | *Oncorhynchus keta* | 3 | 16.6 |
| 8 | Hemoglobin subunit alpha-4-like [*Oncorhynchus keta*] | *Oncorhynchus keta* | 3 | 14 |
| 9 | Hemoglobin subunit alpha-4-like [*Oncorhynchus keta*] | *Oncorhynchus keta* | 4 | 14 |
| 10 | Hemoglobin subunit alpha-like [*Oncorhynchus keta*] | *Oncorhynchus keta* | 1 | 14 |
